# Requirement for Core 2 *O*-Glycans for Optimal Resistance to Helminth Infection

**DOI:** 10.1371/journal.pone.0060124

**Published:** 2013-03-29

**Authors:** Sarah C. Mullaly, Menno J. Oudhoff, Paul H. Min, Kyle Burrows, Frann Antignano, David G. Rattray, Alistair Chenery, Kelly M. McNagny, Hermann J. Ziltener, Colby Zaph

**Affiliations:** 1 The Biomedical Research Centre, University of British Columbia, Vancouver, British Columbia, Canada; 2 Department of Medical Genetics, University of British Columbia, Vancouver, British Columbia, Canada; 3 Department of Pathology and Laboratory Medicine, University of British Columbia, Vancouver, British Columbia, Canada; McGill University, Canada

## Abstract

The migration of lymphocytes to the small intestine is controlled by expression of the integrin α4β7 and the chemokine receptor CCR9. However, the molecules that specifically regulate migration to the large intestine remain unclear. Immunity to infection with the large intestinal helminth parasite *Trichuris muris* is dependent upon CD4^+^ T cells that migrate to the large intestine. We examine the role of specific chemokine receptors, adhesion molecules and glycosyltransferases in the development of protective immunity to *Trichuris*. Mice deficient in expression of the chemokine receptors CCR2 or CCR6 were resistant to infection with *Trichuris*. Similarly, loss of CD34, CD43, CD44 or PSGL-1 had no effect on resistance to infection. In contrast, simultaneous deletion of the Core2 β1,6-*N*-acetylglucosaminyltransferase (C2GnT) enzymes C2GnT1 and C2Gnt2 resulted in delayed expulsion of worms. These results suggest that C2GnT-dependent modifications may play a role in migration of protective immune cells to the large intestine.

## Introduction

Lymphocyte migration to inflamed tissues is a complex, dynamic and highly regulated process. Several distinct families of chemokine receptors, as well as adhesion molecules and glycoprotein modifying enzymes have been implicated in licensing homing to the appropriate inflammatory site. Tissue-specific inflammatory homing is characterized by a remarkably complex interplay between these molecules. For example, it is well known that distinct chemokine receptor expression patterns are observed on cells that migrate to skin versus mucosal sites [1]. In addition, adhesion molecules such as integrins, selectins and selectin ligands can also be dynamically regulated by extrinsic signals that dictate the homing patterns of the responding cells [2]. Finally, post-translational modifications of proteins on the cell surface by enzymes that add carbohydrate residues are also responsive to external stimuli and can promote cellular migration. For example, core 2 *O*-glycosylation catalysed by the β1,6-*N*-acetylglucosaminyltransferase (C2GnT) family is required for modification of PSGL-1 so it can bind selectins, an interaction required for efficient homing of T cells to sites of inflammation [3]. Together, the combinatorial expression of these distinct homing receptors allows for the precise tissue-specific homing patterns observed *in vivo*.

The gastrointestinal tract is a primary site of infection for multiple pathogens. Following infection, dendritic cells (DCs) migrate to the draining mesenteric lymph node where they prime and activate antigen-specific CD4^+^ T helper (T_H_) cells. Activated T_H_ cells then migrate to the intestine where they mediate their effector functions. Signals from intestinal DCs, such as retinoic acid, promote the expression of the intestinal homing molecules CCR9 and α4β7 integrin [4]. It is clear from several studies that these specific molecules are critical for homing of T_H_ cells to the small intestine during infection as well as during the development of oral tolerance [5]. However, the molecules that regulate T_H_ cell homing to the large intestine are less well defined. In mice, resistance to infection of mice with *Trichuris muris*, a helminth parasite of mice that infects the large intestine, is critically dependent upon the migration of T_H_2 cells to the gut where production of IL-4 and IL-13 activate intestinal epithelial cells to promote worm expulsion [6]. Thus, the development of protective immunity to *Trichuris* is strongly indicative of functional T_H_ cell migration to the large intestine. As an example of the specificity of this system, immunity to *Trichuris* occurs independently of β7 integrin expression while, in contrast, immunity to the small intestinal helminth parasite *Trichinella spiralis* is β7 integrin-dependent [7]. It has also been shown that the chemokine CCL2, a ligand for the homing receptor CCR2, is required for immunity to *Trichuris* [8]. Svensson *et al.* further demonstrated that T_H_ cell migration to the large intestine was dependent upon Gαi-coupled receptors, as treatment with pertussis toxin abrogated the accumulation of T_H_ cells in the large intestine [9]. However, since CCL2 has also been shown to directly promote T_H_2 cell responses [10], [11] and since pertussis toxin can induce IL-12 and T_H_1 differentiation [12], it has remained unclear whether defective T_H_ cell migration to the large intestine was directly affected by these treatments. In aggregate, the examples cited here highlight the fact that specific receptors and molecules involved in T_H_ migration to the large intestine have not been identified. In this manuscript, we used the *Trichuris* infection model and several genetically modified mice to elucidate the classes of molecules that are required for homing of T_H_ cells to the large intestine.

Following examination of a wide variety of molecules associated with lymphocyte homing, we demonstrate that several well-characterized receptors and enzymes are completely dispensable for immunity to *Trichuris* infection. In contrast, simultaneous deletion of two members of the C2GnT family (C2GnT1 and C2GnT2) resulted in delayed worm expulsion. Taken together, these results show that while the C2GnT enzymes are partially responsible for some aspects of immunity to *Trichuris* infection, the precise molecular mechanisms of lymphocyte homing to the large intestine remain undefined.

## Materials and Methods

### Ethics Statement

Experiments were approved by the University of British Columbia Animal Care Committee (Protocol number A08-0673) and were in accordance with the Canadian Guidelines for Animal Research.

### Animals, Parasites, Ag and Infections

C57BL/6, RAG1^−/−^, CD44^−/−^ and CCR6^−/−^ mice on a C57BL/6 background were originally obtained from The Jackson Laboratory and were bred in-house. CD34^−/−^ mice have been previously described [13]. CD43^−/−^ mice on a C57BL/6 background have been previously described [14]. C2GnT1^−/−^, C2GnT2^−/−^, C2GnT3^−/−^ and C2GnT1/2/3^−/−^ mice have been described previously [15]. Mice were bred and maintained under specific pathogen-free conditions. Purification of *Trichuris* eggs and antigen was performed as described previously [16]. Mice were orally infected with 200 embryonated eggs and sacrificed 21 or 35 days post-infection.

### Analysis of Trichuris-induced Immunity

Single cell suspensions from mLN of naïve or *Trichuris-*infected mice were plated at 3–4×10^6^/ml in medium or in the presence of antibodies against CD3 (145-2C11) and CD28 (37.51; 1 µg/ml each; eBioscience) for 72 h. Cytokine production from cell-free supernatants was determined by standard sandwich ELISA using commercially available antibodies (eBioscience). *Trichuris*-specific serum IgG1 levels were determined by ELISA on plates coated with *Trichuris* antigen (5 µg/ml).

### RNA Isolation and Quantitative Real-time PCR

RNA was purified from sections of large intestine using mechanical disruption followed by TRIzol according to the manufacturer’s instructions. Reverse transcription was used to generate cDNA and qPCR was performed using SYBR green Quantitect primer sets (Qiagen). Reactions were run on an ABI 7900 real-time PCR machine (Applied Biosystems). Samples were normalized against actin and are expressed as fold over naïve.

### Statistics

Results are presented as mean ± SEM of individual animals. Statistical significance was determined by unpaired Student’s *t*-test (when comparing two samples) or ANOVA with a Bonferonni post-hoc test (when comparing more than 2 samples) using Prism software (GraphPad). Results were considered significant with a *P* value of <0.05.

## Results

### CCR2 is not Required for Immunity to *Trichuris*

**Figure 1:**
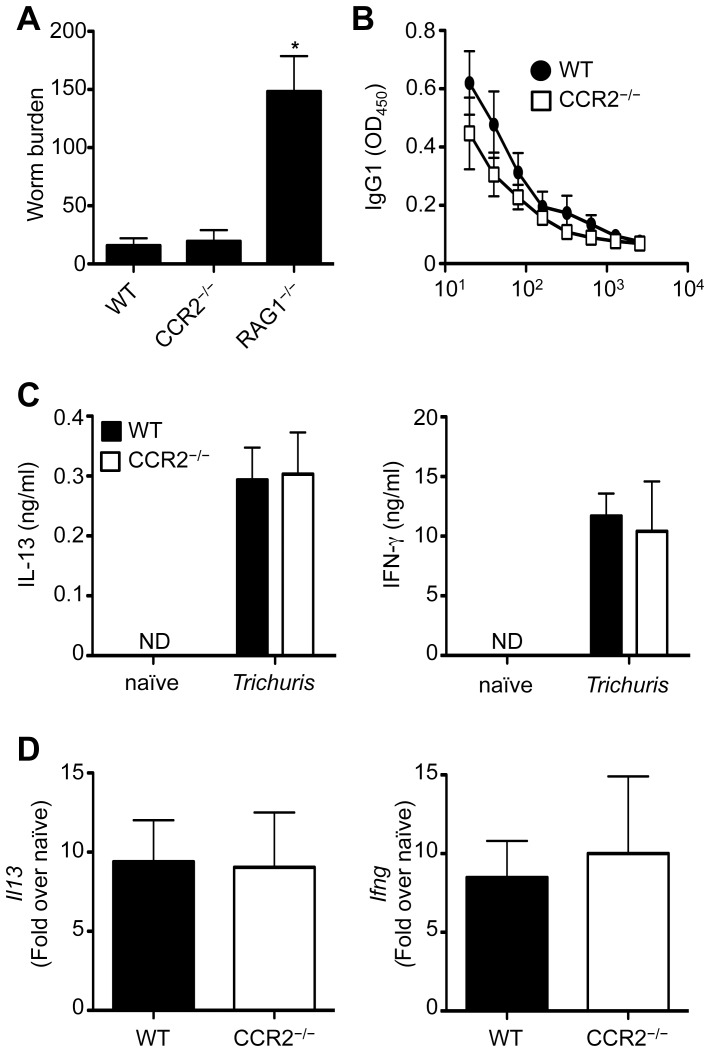
CCR2-deficient mice are resistant to *Trichuris* infection. WT, CCR2^−/−^ and RAG^−/−^ mice were orally infected with 200 *Trichuris* eggs. (**A**) Number of worms per mouse was determined microscopically at day 21 following infection. (**B**) *Trichuris*-specific serum IgG1 levels were assessed by ELISA from 21-day infected WT (•) and CCR2^−/−^ (□) mice. (**C**) mLN cells from WT and CCR2^−/−^ mice were restimulated with anti-CD3/CD28 Abs for 72 h and supernatants were analyzed by ELISA for production of IL-13 and IFN-γ. (**D**) Expression of *Il13* and *Ifng* mRNA levels in the large intestine were assessed by qPCR at day 21 following infection and data are expressed as relative to uninfected control mice. Data in (**A**) are averaged from 3 experiments (n = 6–12); Data in (**B**) to (**D**) are representative of one experiment of 3 independent experiments (n = 6–12).

Previous studies have suggested that CCL2 and its receptor CCR2 are required for immunity to *Trichuris* [8], [9]. To directly test whether CCR2 was required for the development of protective immunity, WT and CCR2^−/−^ mice were infected with *Trichuris*. Similar to WT mice, CCR2^−/−^ mice were resistant to *Trichuris*, expelling almost all worms by day 21, while immunodeficient RAG1^−/−^ mice were unable to eradicate any parasites (**Figure 1A**). *Trichuris*-specific serum IgG1 titers, a hallmark of systemic Th2 cell responses, were similar between WT and CCR2^−/−^ mice (**Figure 1B**). Further, we could not detect any significant differences in the production of IL-13 or IFN-γ by restimulated mesenteric lymph node (mLN) cells or in the expression of *Il13* and *Ifng* in the intestines of infected WT and CCR2^−/−^ mice (**Figure 1C,D**). Thus, CCR2 is dispensable for the development of protective immunity to *Trichuris*.

### Immunity to *Trichuris* is Independent of CCR6

**Figure 2:**
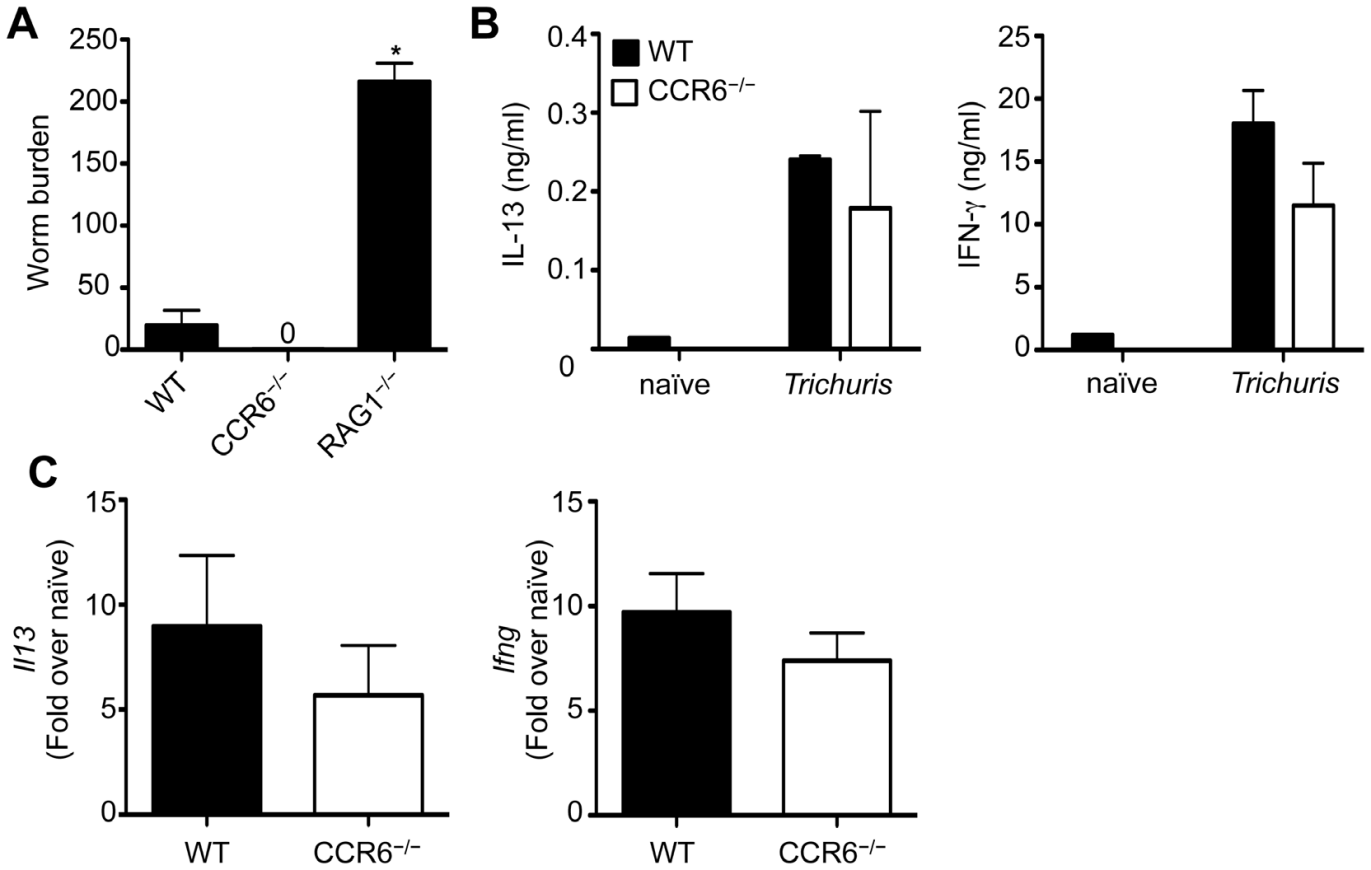
CCR6-deficient mice are resistant to *Trichuris* infection. WT, CCR6^−/−^ and RAG1^−/−^ mice were orally infected with 200 *Trichuris* eggs. (**A**) Number of worms per mouse was determined microscopically at day 21 following infection. (**B**) mLN cells from WT and CCR6^−/−^ mice were restimulated with anti-CD3/CD28 Abs for 72 h and supernatants were analyzed by ELISA for production of IL-13 and IFN-γ. (**C**) Expression of *Il13* and *Ifng* mRNA levels in the large intestine were assessed by qPCR at day 21 following infection and data are expressed as relative to uninfected control mice. Data in (**A**) are averaged from 2 experiments (n = 4–8); Data in (**B**) and (**C**) are representative of one experiment of 2 independent experiments (n = 4–8).

We next examined whether other chemokine receptors may be required for immunity to *Trichuris*. We focused on CCR6, as this receptor is expressed on T_H_ cells and has been implicated in other intestinal immune responses [17]. However, following infection with *Trichuris*, CCR6^−/−^ mice were able to completely clear their worm burdens (**Figure 2A**). Other parameters of immunity including production of IL-13 and IFN-γ by restimulated mLN cells (**Figure 2B**) or expression of *Il13* and *Ifng* in the intestine (**Figure 2C**) were equivalent between WT controls and CCR6^−/−^ mice. Thus, expression of CCR6 is not required for immunity to *Trichuris*.

### Adhesion Molecules CD34, CD43, CD44 and PSGL-1 are also not Essential for Immunity to *Trichuris*

**Figure 3:**
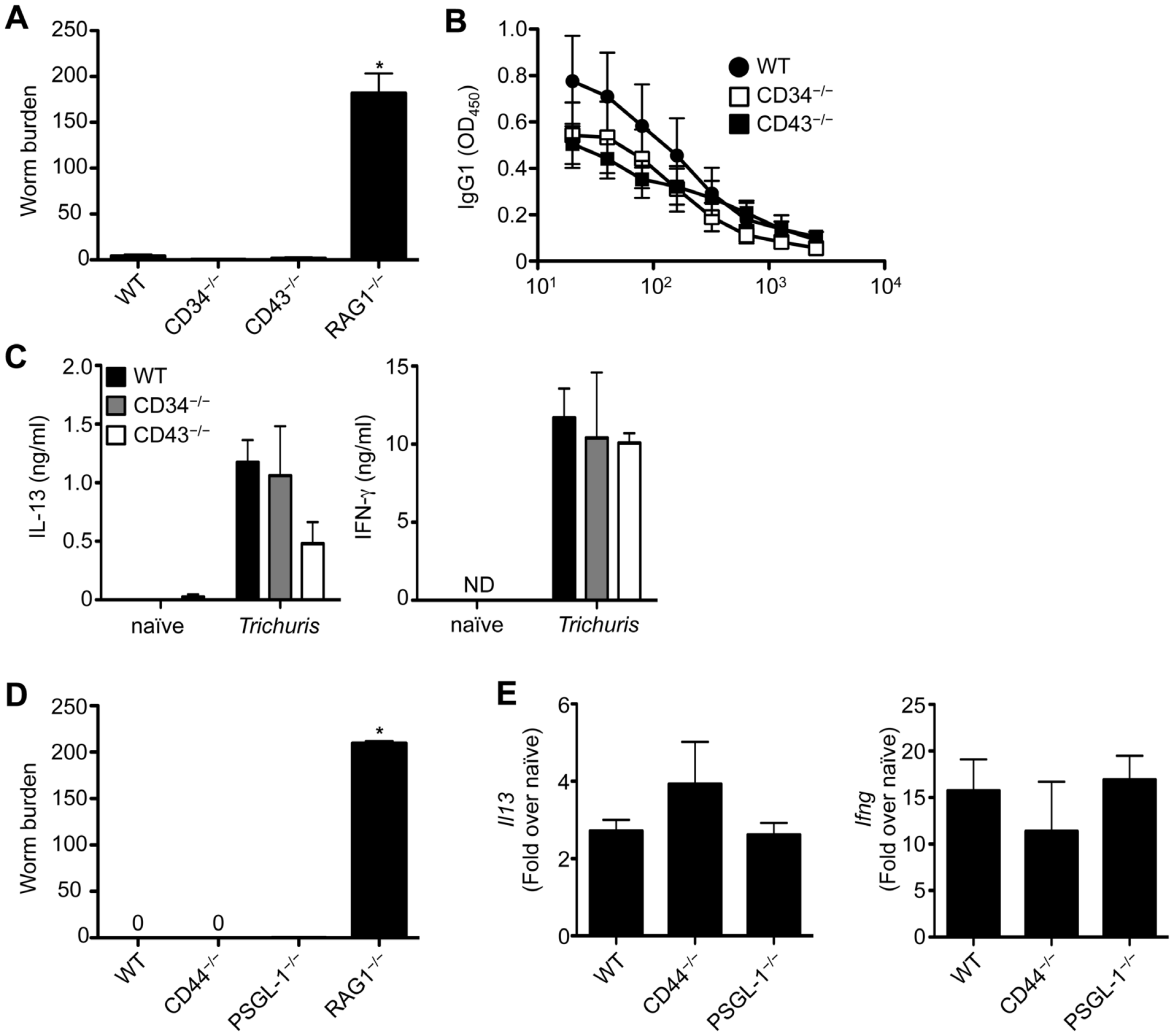
CD34, CD43, CD44 and PSGL-1 are dispensable for resistance to *Trichuris*. WT, CD34^−/−^, CD43^−/−^, CD44^−/−^, PSGL-1^−/−^ and RAG1^−/−^ mice were orally infected with 200 *Trichuris* eggs. (**A**) and (**D**) Number of worms per mouse was determined microscopically at day 21 following infection. (**B**) *Trichuris*-specific serum IgG1 levels were assessed by ELISA from 21-day infected WT (•), CD34^−/−^ (□) and CD43^−/−^ (**▪**) mice. (**C**) mLN cells from WT, CD34^−/−^ and CD43^−/−^ mice were restimulated with anti-CD3/CD28 Abs for 72 h and supernatants were analyzed by ELISA for production of Il-13 and IFN-γ. (**E**) Expression of *Il13* and *Ifng* mRNA levels in the large intestine were assessed by qPCR at day 21 following infection and data are expressed as relative to uninfected control mice. Data in (**A**) and (**D**) are averaged from 2 experiments (n = 4–8); Data in (**B**), (**C**) and (**E**) are representative of one experiment of 2 independent experiments (n = 4–8).

We have previously shown that the integrin CD103 is not required for immunity to *Trichuris* [16]. However, the role of other well-established adhesion molecules during *Trichuris* infection has not been examined. CD34 and CD43 are two distantly related sialomucins that are differentially and dynamically expressed on a wide variety of immune cells [18], [19]. CD34^−/−^ mice display increased resistance to a wide variety of inflammatory diseases including allergic lung inflammation, arthritis and *Salmonella* infection [20]–[23], and CD43 has been shown to regulate T_H_ cell migration *in vivo* [24]. Both CD34^−/−^ or CD43^−/−^ mice displayed a resistant phenotype following infection with *Trichuris*, as measured by worm burden (**Figure 3A**), immunoglobulin production (**Figure 3B**) and cytokine production (**Figure 3C**). Thus, the sialomucins CD34 and CD43 are not critical components of the molecular machinery controlling the migration of protective T_H_ cells to the large intestine.

T_H_ cell migration is also regulated by expression of CD44 and PSGL-1 [25], [26]. CD44 is the receptor for low molecular weight hyaluronan, a marker of inflamed tissues [26]. Naïve T_H_ cells express low levels of CD44 that increase upon T_H_ cell activation [27]. In contrast, PSGL-1 is constitutively expressed on T_H_ cells but is post-translationally modified by several distinct glycosyltransferases expressed in activated T_H_ cells that then endow it with the ability to bind to P-selectin that is expressed on the luminal surface of inflamed endothelial cells [25]. Similar to results obtained above, CD44^−/−^ and PSGL-1^−/−^ mice were also resistant to *Trichuris* infection, clearing worms by day 21 (**Figure 3D**) and expressing equivalent levels of *Il13* and *Ifng* (**Figure 3E**). These results demonstrate that surprisingly, canonical adhesive receptors are also not required for the development of protective immunity to *Trichuris*.

### C2GnTs are Required for Optimal Immunity to *Trichuris*

**Figure 4:**
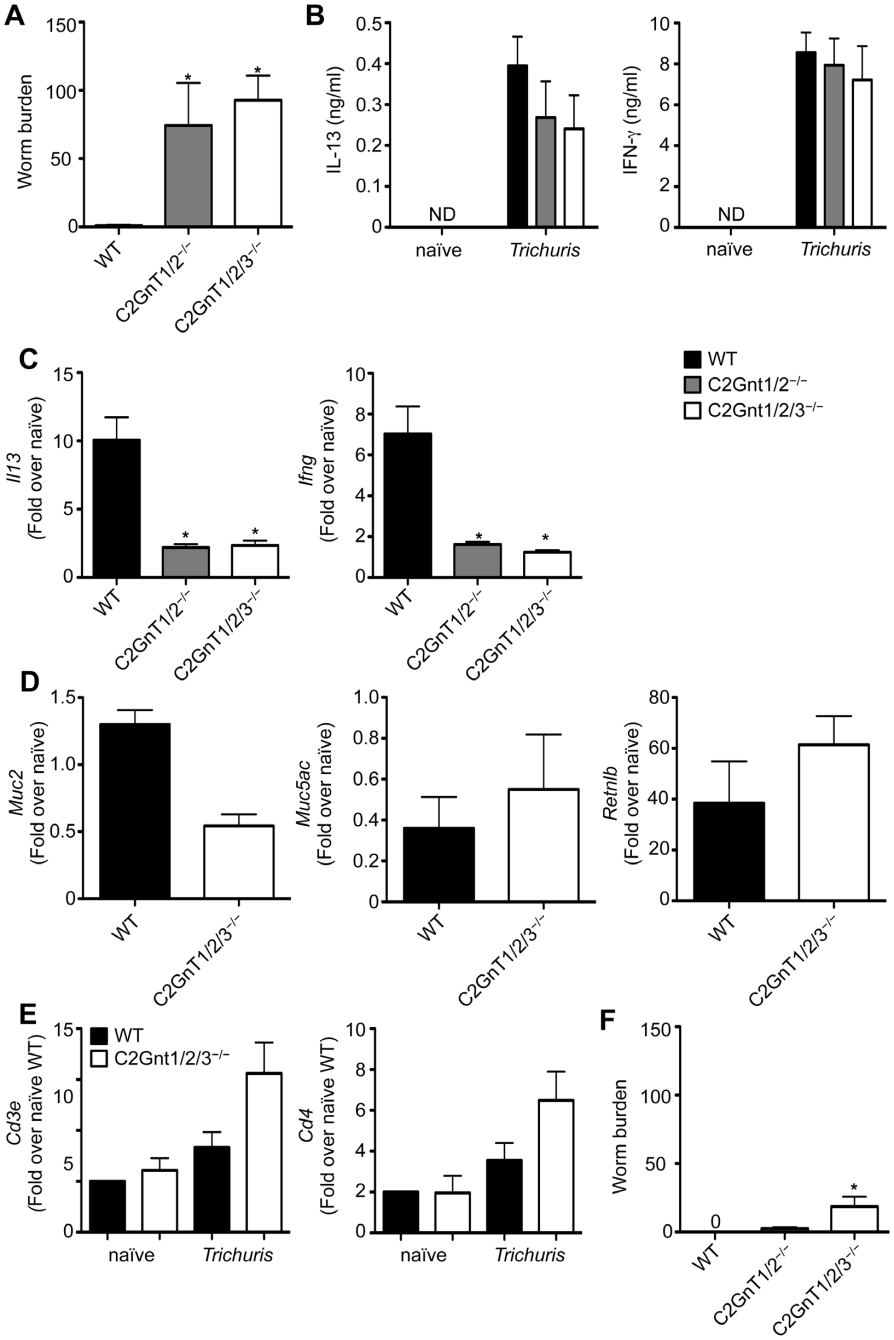
C2GnT1/2/3^−/−^ mice display delayed parasite clearance following infection with *Trichuris*. WT, C2GnT1/2^−/−^ and C2GnT1/2/3^−/−^ mice were orally infected with 200 *Trichuris* eggs. (**A**) and (**F**) Number of worms per mouse was determined microscopically at day 21 (**A**) and day 35 (**D**) following infection. (**B**) mLN cells from WT, C2GnT1/2^−/−^ and C2GnT1/2/3^−/−^ mice were restimulated with anti-CD3/CD28 Abs for 72 h and supernatants were analyzed by ELISA for production of IL-13 and IFN-γ. (**C**) to (**E**) Expression of *Il13* and *Ifng* (**C**), *Muc2*, *Muc5ac* and *Retnlb* (**D**) or *Cd3e* and *Cd4* (**E**) mRNA levels in the large intestine were assessed by qPCR at day 21 following infection and data are expressed as relative to uninfected control mice. Data in (**A**) and (**F**) are averaged from 4 experiments (n = 8–16); Data in (**B**) to (**E**) are representative of one experiment of 4 independent experiments (n = 8–16).

Selectin ligand formation on PSGL-1 requires core 2 protein *O*-glycosylation, a post-translational modification that is exclusively catalyzed by the family of β1,6-*N*-acetylglucosaminyltransferases (C2GnT). While C2GnT1 has been firmly established to form selectin binding sites, it is not yet clear whether and to what degree the other two members of this enzyme family, C2GnT2 and C2GnT3, contribute in a physiological setting to selectin ligand formation [15], [28]. C2GnT1 and C2GnT3 are expressed primarily by lymphocytes while C2GnT2 is associated with goblet cell mucin production in the intestinal epithelium. Single deletion of any of these enzymes (C2GnT1^−/−^, C2GnT2^−/−^ or C2GnT3^−/−^ mice) and double deletion of C2GnT1 and C2GnT3 (C2GnT1/3^−/−^ mice) had no effect on immunity to *Trichuris* (**data not shown**). Surprisingly, mice doubly-deficient in C2GnT1 and C2GnT2 (C2GnT1/2^−/−^ mice) and mice deficient in all 3 C2GnT family members (C2GnT1/2/3^−/−^ mice) were unable to expel their worms by day 21 post-infection (**Figure 4A**). Strikingly, this susceptibility was not associated with dysregulated IFN-γ or IL-13 production by mLN cells (**Figure 4B**), suggesting that priming of T_H_ cell responses in the draining mLN was unaffected. Importantly, we observed decreased levels of the cytokines *Il13* and *Ifng* in the intestine (**Figure 4C**). However, expression of intestinal epithelial cell-specific effector molecules such as Muc2, Muc5ac and RELM-β were not significantly different between WT and C2GnT1/2/3^−/−^ mice (**Figure 4D**). These results indicate that in the absence of the core 2 *O*-glycosylases there is either impaired effector T_H_ cell migration to the large intestine or a failure to produce cytokines at the site of infection. Indeed, we failed to detect any defects in expression of the T_H_ cell-specific molecules *Cd3e* and *Cd4* in the intestines of infected C2GnT1/2/3^−/−^ mice (**Figure 4E**), demonstrating that defective T_H_ cell homing to the infected tissue is not likely the cause of the inability to expel worms by day 21 post-infection in the C2GnT1/2/3^−/−^ mice. Consistent with this, analysis of worm burdens at day 32 demonstrated that both C2GnT1/2^−/−^ and C2GnT1/2/3^−/−^ mice were eventually able to significantly reduce worm burden, albeit slower than WT mice (**Figure 4F**). Taken together, our results demonstrate that protective immunity to *Trichuris* is partially mediated by expression C2GnT enzymes.

## Discussion

We demonstrate that several chemokine receptors and adhesion molecules are dispensable for large intestinal immune responses *in vivo*. These results are surprising and suggest that other mechanisms are in place for the development of immunity in the large intestine.

We show that CCR2 and CCR6 are not required for a protective immune response to *Trichuris*. It is possible that other intestinal-tropic chemokine receptors such as CCR9, which has been shown to target lymphocytes to the small intestine [29], may play a role in homing of T_H_ cells to the large intestine. Indeed, CCR9 has recently been demonstrated to regulate disease development in a chemically-induced model of colitis [30]. While this model can develop independently of T_H_ cells, it provides an intriguing potential mechanism that should be tested.

The exact role of the sialomucins CD34 and CD43 in lymphocyte trafficking is unclear. CD34 deficiency results in heightened resistance to a variety of inflammatory diseases due to defects in migration of many cell types including mast cells [31], dendritic cells [20], [32] and granulocytes [23], [33]. CD43 has been shown to regulate T_H_ cell migration to lymph nodes [24]. However, as both of these proteins are dispensable for immunity to *Trichuris*, it is likely that they play no role in the migration of large intestinal-tropic T_H_ cells.

PSGL-1 is a central player in the recruitment of T cells to sites of inflammation and P-selectin ligand formation on PSGL-1 is believed to be dependent on C2GnT1 enzyme activity [34], [35]. The fact that both C2GnT1 and PSGL-1 are not required for immunity to *Trichuris*, firmly rules out involvement of the PSGL-1/P-selectin axis in recruitment of protective T_H_ cells during large intestinal immune responses. Interestingly, loss of C2GnT1 combined with loss of C2GnT2 enzymes, or loss of all three enzymes leads to a delay in worm clearance, whereas single deletion of any of the C2GnT enzymes and double deletion of C2GnT1 with C2GnT3 has no effect on immunity. Delayed worm clearance was associated with reduced intestinal cytokine responses, suggesting that combined lack of C2GnT1 and C2GnT2 resulted in reduced recruitment of inflammatory cells to the colon. However, we failed to observe decreased expression of *Cd3e* and *Cd4* in the intestinal tissue of naïve or infected C2GnT1/2/3^−/−^ mice, suggesting that T cell migration was not impaired. Thus, it is possible that other cell types required for optimal T cell cytokine production require C2GnTs for their homing to the intestine. While a main function of C2GnT1 enzyme is seen in the control of leukocyte homing in inflammation [34], there is so far no evidence that C2GnT2 can contribute to tissue homing receptor expression. Nevertheless, it is possible that C2GnT2 might contribute to T_H_ cell homing and that this becomes relevant in absence of the PSGL-1/P-selectin axis. Identification of such a ligand may provide a potential marker of T_H_ cells that have the ability to migrate to the large intestine.

Alternatively, C2GnT2 is primarily associated with mucin production by goblet cells in the intestine and loss of this enzyme has been shown to be associated with increased sensitivity to colitis [15]. Decreased resistance to *Trichuris* infection might thus be due to a combination of a subtle T_H_ homing defect associated with loss of C2GnT1, and subtle defects in C2GnT2^−/−^ mice associated with reduced mucosal function due to altered mucin glycosylation. In support of this latter scenario is the observation that intestinal epithelial cell-dependent expression of the mucins Muc2 [36] and Muc5ac [37] are critical for immunity to *Trichuris*.

In summary, we have demonstrated that C2GnT enzymes are required for the optimal development of mucosal T cell immunity in the large intestine during helminth infection. Our results suggest that other not yet identified C2GnT substrates may regulate intestinal immune responses.
